# The immunology of SARS-CoV-2 infections and vaccines

**DOI:** 10.1016/j.smim.2020.101422

**Published:** 2020-08

**Authors:** Lilit Grigoryan, Bali Pulendran

**Affiliations:** Institute for Immunology, Transplantation and Infectious Diseases, Department of Pathology, Department of Microbiology & Immunology, Stanford University School of Medicine, Stanford, CA, 94305, United States

**Keywords:** COVID-19, Vaccines, Systems vaccinology

## Abstract

SARS-CoV-2, the virus that causes COVID-19, emerged in late 2019, and was declared a global pandemic on March 11th 2020. With over 50 million cases and 1.2 million deaths around the world, to date, this pandemic represents the gravest global health crisis of our times. Thus, the race to develop a COVID-19 vaccine is an urgent global imperative. At the time of writing, there are over 165 vaccine candidates being developed, with 33 in various stages of clinical testing. In this review, we discuss emerging insights about the human immune response to SARS-CoV-2, and their implications for vaccine design. We then review emerging knowledge of the immunogenicity of the numerous vaccine candidates that are currently being tested in the clinic and discuss the range of immune defense mechanisms that can be harnessed to develop novel vaccines that confer durable protection against SARS-CoV-2. Finally, we conclude with a discussion of the potential role of a systems vaccinology approach in accelerating the clinical testing of vaccines, to meet the urgent needs posed by the pandemic.

## 1 Warp speed vaccines: the global race to develop a COVID-19 vaccine

**Fig. 1:**
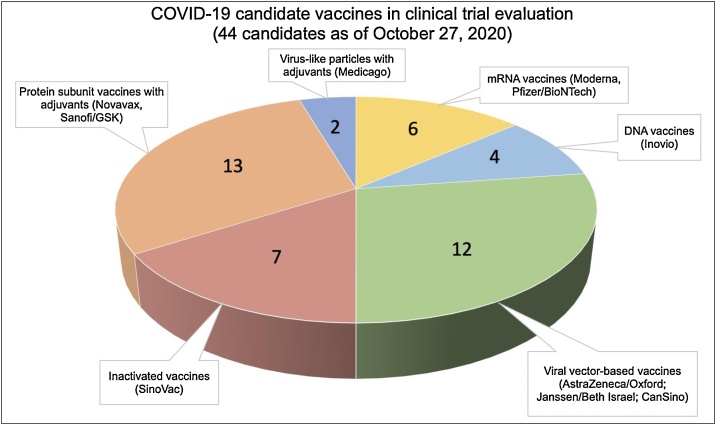
COVID-19 vaccine candidates in clinical evaluation to date. Numbers represent the number of vaccine candidates of that platform technology currently in clinical testing.

**Fig. 2:**
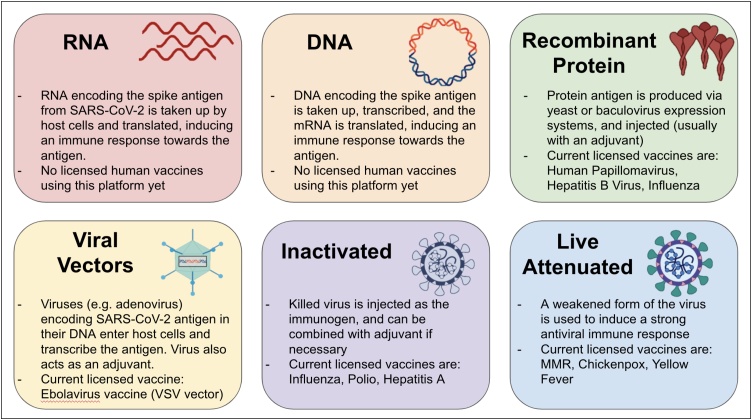
Current vaccine platforms of COVID-19 vaccine candidates.

At the present time, there are over 187 vaccine candidates being developed against COVID-19, with 44 at various stages of human clinical trial testing (Fig. 1) [1]. At the time of writing, the early results from early-phase clinical trials testing 9 vaccine candidates have been released. The vaccines tested represent a diverse range of platform technologies (Fig. 2) including mRNA vaccines from Moderna [2] and Pfizer/BioNTech [3], adenoviral vector-based vaccines from CanSino [4], Oxford [5], the Gamaleya Research Institute from Russia [6] and Janssen/Beth Israel [7], an adjuvanted recombinant protein vaccine from Novavax [8] and two inactivated virus vaccines [9,10]. The results from the early phase clinical trials of these vaccine candidates are discussed below. In general, the results from these trials suggest that vaccination was relatively safe and reasonably well tolerated. Importantly, all vaccination modalities induced detectable ELISA binding Ab titers, as well as varying levels of neutralizing Ab titers. The encouraging results of these studies are discussed in detail below, but despite the unprecedented pace (“warp speed”) at which these vaccines are being developed, it remains to be seen in phase 3 efficacy trials, how effective these vaccines will be. Phase 3 trial efficacy data from some of the leading vaccine candidates are expected within the next several weeks to months.

However, the vast majority of these vaccine efforts are predicated on the idea of inducing neutralizing antibodies (nAb), but a major challenge is to induce a high magnitude and durability of nAb titers. Furthermore, it is not known what magnitude of nAb is needed for protection, or what the durability of the nAb responses would be. Therefore, several efforts are underway to assess adjuvants to induce enhanced and durable nAb responses to lead COVID-19 vaccine candidates. In addition, there are major obstacles with regards to the current testing strategies that advance vaccines into clinical trials. The traditional vaccine development pipeline involving preclinical testing in mice and non-human primates, followed by testing in humans in phase 1, 2, 3 clinical trials, can typically take several years, sometimes up to 15 years, and cost several hundred million dollars per vaccine [11,12]. Thus, this traditional pipeline is untenable when evaluating a plethora of vaccine concepts to meet the urgent needs posed by the pandemic and needs to be modified in order to fulfil the current high demand. To this end, recent advances in systems vaccinology provide a strategy to identify signatures of vaccine immunity and efficacy that can be used to screen many COVID-19 vaccine concepts prior to their advancements into larger phase 2 and phase 3 trials. In this review, we discuss the known facets of natural and vaccine-induced immunity and offer some immunological concepts that need to be considered for the rational design of current and future vaccines against COVID-19. First, we discuss the evidence for natural immunity induced by SARS-CoV-2 infection, with a focus on humoral and T cell responses. Next, we evaluate the humoral and cellular immune responses induced by the vaccine candidates tested in human clinical trials, followed by a discussion on protection from infection based on pre-clinical studies in non-human primates. Next, we discuss how other immunological concepts, such as resident memory T cells and trained immunity could be harnessed to induce more synergistic and robust immune response to infection. In addition, we discuss the potential of adjuvants in improving vaccine-induced immunity. Lastly, we end with a discussion of how systems biology techniques can be used to accelerate the vaccine testing pipeline and help identify more candidates with high potential for efficacy and safety.

## 2 Infection-induced immunity against SARS-CoV-2

Infection of a person with a pathogen often results in an imprint of that infection on the immune system, a phenomenon known as immunological memory, that can protect that person from a subsequent infection for decades. This occurs through the induction of antigen specific memory B and T lymphocytes, as well as a durable antibody response, which prevents reinfection. Thus, natural immunity is a feature of many viral infections, such as measles, mumps, chickenpox, which induce neutralizing antibody responses that are remarkably stable, with half-lives ranging from an estimated 50 years for varicella-zoster (chickenpox), 92 years for vaccinia, 542 years for mumps and greater than 3000 years for measles [13]. However, in the case of SARS-CoV-2, the extent to which infection can protect against subsequent reinfection is unclear. In addition, if there is indeed protection against reinfection, how long lasting it is, is currently a topic of intense discussion. Below, we present the evidence of naturally-acquired immunity from non-human primate studies of SARS-CoV-2 infection, human challenge models with other coronaviruses, and human studies of SARS-CoV-2 infected patients.

### 2.1 Evidence from nonhuman primate studies

Two independent studies involving rhesus macaques showed that infection with SARS-CoV-2 protected the animals against reinfection [14,15]. However, the secondary challenge in these studies was performed 4 weeks post primary infection, which raises the question of how durable this protection was. In addition, it is important to note that the latter study investigated the viral load in both nasal swabs and BAL [15], and observed that while there was a profound 4-logarithmic-fold reduction in viral RNA in the BAL of monkeys who had previous infection compared to controls, the differences in viral load in the nasal swab, although statistically significant, were not as profound (at most 2-log-fold), suggesting that protection is not sterilizing. Taken together, the results from the non-human primate studies provide evidence for significant (albeit not sterilizing) protection against reinfection of SARS-CoV-2, although the extent to which infection induces durable protection remains unknown.

### 2.2 Evidence from human challenge studies with other coronaviruses

**Fig. 3:**
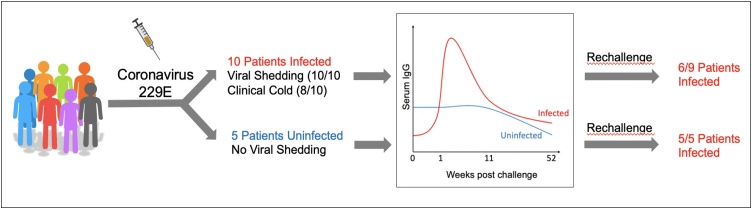
Human challenge study of coronavirus 229E (Callow, 1990 [22]). 15 volunteers were inoculated with 229E, 10 of whom became infected. Chart shows average antibody titers in the infected and infected groups. All subjects were rechallenged, and all originally uninfected patients got infected upon secondary exposure, compared to 6 out of the 9 patients who were originally infected.

In considering natural immunity to SARS-CoV-2, it is instructive to draw from prior knowledge of the immune response to other coronaviruses including SARS-CoV, MERS, and some of the common cold viruses that infect humans (such as 229E and OC43). Although each of these viruses are unique, information about the immune responses against one virus could offer insights into the mechanisms of protection against SARS-CoV-2. Studies in which humans have been experimentally infected with such viruses (“*C*ontrolled *H*uman Infection Models” or CHIM) are of particular relevance, since these can control for the duration of time between the primary infection and the rechallenge, as well as the baseline immune features (such as pre-infection antibody titers) in participants [[16], [17], [18], [19], [20], [21]]. In a CHIM study of HCoV-229E [22], a coronavirus that causes a common cold, 15 patients were inoculated with the virus, 10 of whom got infected upon exposure (Fig. 3). Interestingly, the infected group had slightly lower baseline virus-specific binding titers of serum IgG compared to the uninfected group (∼3.5 log_10_ units in uninfected group versus 3.0 log_10_ units in the infected group). Upon primary infection, serum IgG titers in infected patients significantly increased, but gradually waned over the course of a year, declining to the pre-vaccination titers detected in the group that failed to get infected. Most importantly, a rechallenge with homologous virus after 1 year of primary infection resulted in infection of all of the patients in the originally uninfected group, and 6/9 patients (i.e. 67 %) in the infected group (Fig. 3). A similar study, however, showed complete protection in all 6 of the volunteers that were initially challenged with a 229E-related isolate labeled as TO, and rechallenged 1 year post primary infection [23]. However, interestingly, when an additional 8 volunteers were initially inoculated with 229E or related strains, and subsequently reinoculated 8–14 months later with a heterologous 229E strain virus, 5 out of these volunteers developed cold symptoms, indicating that protection against heterologous strains was weaker. Thus, the human challenge studies with the 229E virus show that some protection exists for this particular coronavirus, although not all patients are protected from reinfection, and this protection may not be durable, and may depend on the similarity of the strains of the 229E virus. However, the extent to which these results can be extrapolated onto SARS-CoV-2 immunity remains to be investigated. In particular, there has been recent discussion about prospects for a human challenge model for SARS-CoV-2 [24], which could likely accelerate the pace of vaccine development. However, establishing such a model has serious ethical implications and any efforts of a COVID-19 CHIM study should be discussed rigorously, amongst scientists, clinicians and ethicists.

### 2.3 Evidence from studies of COVID-19 patients

The question of whether there can be reinfection with SARS-CoV-2, after recovery from an initial bout of infection, goes to the heart of the issue of whether the immune system can remember its encounter with this pathogen. A priori, there is no reason to expect that reinfections will not occur, since reinfections have been demonstrated with other respiratory infections, such as influenza or the common cold virus [25,26]. In the case of COVID-19, a study of patients in China detected SARS-CoV-2 by PCR in a small number of patients that were thought to have recovered from COVID-19 weeks before [27]. Furthermore, a recent study found that nasopharyngeal swab PCR-positivity can remain in patients weeks after resolution of symptoms [28], and in pediatric cases, fecal virus shedding long after nasopharyngeal negative swabs has been observed [29]. Such studies have raised the possibility of reinfections. However this issue has been difficult to establish, because viral RNA was not sequenced and therefore the identities and putative relationship between the viruses that caused primary and secondary infections are unknown. Furthermore, the RNA was detected only a few weeks following the primary infection and live virus was not recovered, therefore it is unclear whether they represent reinfections or merely residual RNA from the prior bout of infection. Two recent studies, have however, provided strong evidence for reinfection of two patients, one in Hong Kong and the other in Nevada [30,31]. In both of these cases, the virus from nasopharyngeal swabs was sequenced, and these sequences differed to such an extent that the virus isolated from reinfection was designated as belonging to a different clade to the ones isolated from the prior infections, providing evidence that these cases did indeed represent reinfections. In the case of the Hong Kong patient, reinfection was observed 142 days following the primary infection, and was asymptomatic [31]. Thus, although the primary infection did not induce sterilizing protection, it may have protected the patient against severe disease. In the case of the Nevada patient, the secondary infection occurred about a month after recovery from the first infection, and in this case, the patient had quite severe disease [30]. Thus, there are as yet two cases of SARS-CoV-2 reinfections in patients. Considering that there have so far been some 45 million cases of COVID-19 worldwide, one wonders how frequent such reinfection events might be. As stated above, a priori there is no reason to exclude the possibility of reinfections, although the extent to which they occur remains unknown. It is conceivable that reinfections may be more frequent than anticipated but remain undetectable because in the vast majority of cases they cause asymptomatic infections. Although it is unclear whether SARS-CoV-2 infection can induce protective, long-lasting immunity, we know that SARS-CoV-2 infection induces a myriad of immune responses in humans [32]. The parameters that define effective protective immunity against SARS-CoV-2 are currently unclear. In particular, the contribution of neutralizing antibodies, versus T cells or innate responses needs to be delineated. Below, we discuss the components of immunity that may contribute to protection, particularly, the extent to which these different components of immunity are induced, what their evidence towards protection is, and how durable these responses are.

## 3 The immune response to SARS-CoV-2 infection

### 3.1 Innate immunity to SARS-CoV-2 infection

**Fig. 4:**
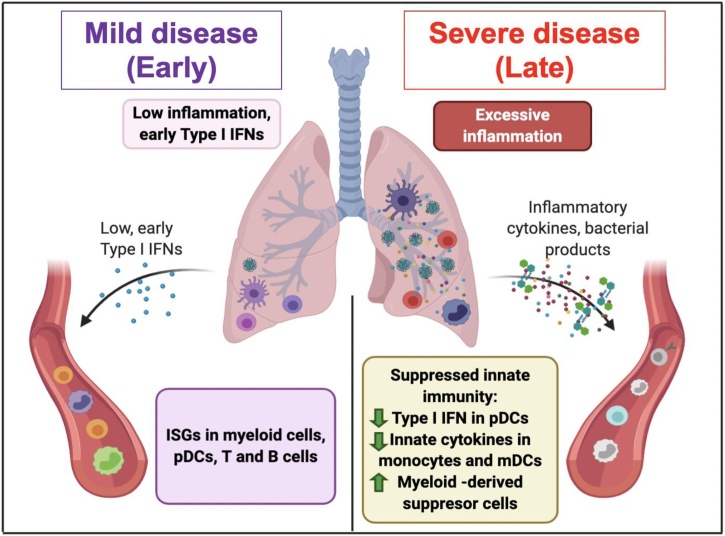
The innate immune response in mild and severe COVID-19 patients. Severe COVID-19 cases appear to have refractory innate immune responses in blood, suggesting a spatial dichotomy in the innate immune response, whereby peripheral innate cells can be inhibited in the face of inflammatory responses reported in the lungs.

Several groups have analyzed cytokine levels in plasma or sera of COVID-19 patients and demonstrated elevated levels of proinflammatory cytokines such as IL-6 and TNFα and their strong association with disease severity, leading to the concept of a cytokine storm [[33], [34], [35], [36], [37], [38], [39], [40]]. Several recent studies have undertaken a deep immune profiling of COVID-19 patients to delineate mechanisms of host immunity [34,37,38,[41], [42], [43]]. A recent study from our group used systems biology tools to address differences between mild versus severe COVID-19 patients [34], where we found increased levels of proinflammatory cytokines such as IL6, MCP-1, CXCL10 in the blood of COVID-19 patients, as well as upregulation of LIGHT (TNFSF14), EN-RAGE, and oncostatin-M, which correlated strongly with the severity of disease. Interestingly, it was previously shown in mice that blocking signaling via lymphotoxin β receptor, a receptor that binds LIGHT, reverses virus-induced systemic shock and respiratory failure [44], while RAGE is a major mediator of pulmonary inflammation [45]. This suggests that these molecules may represent therapeutic targets for COVID-19. In addition, we observed a transient but blunted increase in IFNα in the first two weeks of infection, as observed by several other groups [37,41,43,46]. In addition, we observed reduced frequencies of plasmacytoid DCs, and diminished production of IFNα by plasmacytoid DCs, and proinflammatory cytokines by myeloid DCs, in response to stimulation ex vivo, consistent with results by Zhou et al. [43]. Lastly, increased bacterial products such as LPS and bacterial DNA in blood correlated with disease severity, consistent with the study by Srisawat and colleagues [47]. The concentration of LPS and bacterial DNA was strong correlated with the levels of proinflammatory cytokines such as IL-6, TNF, EN-RAGE, OSM and LIGHT, consistent with the study by Srisawat and colleagues [47], suggesting a potential role for such products in contributing to enhanced inflammatory cytokine production in severe disease [34]. Taken together, SARS-CoV-2 infection appears to induce a transient, but weak type I IFN responses and in severe COVID-19 patients, the innate immune responses in the blood appear to be refractory. Consistent with this refractory state, we and others observed enhanced frequencies of HLA-DR^low^ CD14+ monocytes that expressed EN-RAGE [34,48,49], consistent with recent a phenotype that has been attributed to myeloid-derived suppressor cells [50]. Taken together, these results suggest that SARS-CoV-2 infection results in a spatial dichotomy in the innate immune response, characterized by suppression of peripheral innate immunity in the face of proinflammatory responses that have been reported in the lungs. The consequences and durability of this immune suppression is unclear, but it is conceivable that it may confer enhanced susceptibility to blood borne infections such as dengue. The evolutionary significance of this spatially dichotomous immunity to COVD-19 may represent a homeostatic mechanism aimed at preserving the systemic integrity and survival of the host, in the face of rampant inflammation in the tissue (Fig. 4), since such a systemic immune suppression has previously been observed with sepsis [51,52].

### 3.2 Antibody responses in SARS-CoV-2 infection

Historically, vaccine-induced protective immunity has been largely attributed to the function of antibodies, specifically neutralizing antibodies (nAbs), which block the entry of the virus into target host cells, thus preventing infection. Because of their ability to provide immediate protection upon exposure, the elicitation of nAbs have long been the primary goal of vaccination against many pathogens, including SARS-CoV-2. In line with this, a major focus has been placed on understanding the magnitude, protective capacity and durability of antibody responses in humans infected with SARS-CoV-2. Several studies have reported seroconversion in COVID-19 patients [28,[53], [54], [55], [56]]. A recent study on 1343 COVID-19 patients showed that over 99 % of PCR-positive patients develop anti-spike binding antibodies [28]. Interestingly, a separate study involving 19,860 recovered seropositive individuals showed that the majority (about 70 %) of these individuals have “moderate or high” binding titers, where moderate titers were subjectively defined to be 1:320, while high titers ranged from 1:960 to 1:2880 and above (antibody titers were measured at least 10 days post symptom onset) [55]. Moreover, it was shown that 90 % of seroconverters make neutralizing antibodies (nAbs) and the anti-spike binding antibody titers correlated with SARS-CoV-2 neutralization. In another study of 175 individuals recovered from mild COVID-19, it was shown that 30 % of the patients did not develop high neutralization titers (defined as ID_50_ < 500) [57]. Nevertheless, the nAb titers correlated moderately with RBD binding titers, indicating that high binding antibody titers from previous studies may be indicative of the neutralization capacity of antibodies in these patients. Overall, these data suggest that the majority of COVID-19 patients seroconvert, with most of them producing neutralizing antibodies, but the precise titer of nAb required for protection against reinfection needs to be determined. In this regard, a recent study of people in a fishery vessel outbreak showed that although over 85 % of the people who were on the vessel got infected, the only 3 individuals who had high nAb titers (titers of 161, 171, and 3082) prior to boarding the ship remained uninfected, resulting in a significant correlation of nAbs with protection [58]. However, this study contained only 3 people in the pre-infection nAb group, thus, further studies correlating nAbs with protection are warranted.

In order to investigate the protective capacity of nAbs against SARS-CoV-2, several groups have isolated clones of potently neutralizing antibodies from patients [[59], [60], [61], [62], [63]], and two of these studies showed that transfer of these neutralizing antibodies into rodents conferred protection [62,63], highlighting the role of nAbs against protection in small animal models. Moreover, in a study of a DNA vaccine candidate in NHPs, nAbs were shown to be negatively correlated with viral load in the bronchoalveolar lavage fluid (with an R=-0.77), suggesting nAbs as a correlate of protection [64]. Interestingly, the correlation was weaker for the viral load in the nasal swab, indicating that serum nAb titers may contribute to protection from severe disease in the lower respiratory tract, but might not significantly improve the infection in the upper respiratory tract and viral shedding. Lastly, an mRNA vaccine candidate reviewed below [65] also showed strong negative correlation of nAb titers and protection in NHPs, strengthening the evidence for nAbs as a correlate of protection against SARS-CoV-2.

### 3.3 Durability of antibody responses in SARS-CoV-2 infection

Taken together, the aforementioned studies provide evidence for antibody-mediated protection, but a key question concerns the durability, or lack thereof, of such responses. Thus, several recent studies investigated the durability of the antibody response (Table 1). In one study performed in China, antibody titers in 37 symptomatic versus 37 age and sex-matched asymptomatic PCR-positive individuals were measured during acute disease versus the convalescent phase [66]. Forty percent of the asymptomatic subjects became seronegative during the early convalescent phase (as compared to 12.9 % of patients becoming seronegative in the symptomatic group). In addition, they reported a decrease in the serum IgG 8 weeks after patients were discharged from the hospital, with a median of 71.1 % decrease in titers in asymptomatic patients, and 76.2 % decrease in symptomatic patients. Thus, this study indicated a decline in antibody titers over the course of 8 weeks following resolution of symptoms. In addition, a recent report by Ibarrodo et al. [67] described a study in the United States, involving 34 participants, 30 of whom had confirmed cases of mild COVID-19 infections, and the remaining 4 had cohabited with COVD-19 infected subjects, and had experienced mild COVID-19 like symptoms, but had not been tested because of limited availability of testing. Consistent with the study of Long et al. [66], the authors performed a longitudinal analysis of anti-RBD IgG for 90 days, using ELISA [67], and estimated the half-life of antibodies to be 36 days, and concluded that there was a “rapid decay” of humoral immunity to SARS-CoV-2. Furthermore, Ma et al. assessed the RBD-specific binding antibody titers in 27 COVID-19 convalescent patients, over 28−99 days after hospital discharge [68]. Almost all patients had severe or moderate COVID-19 and had high titers of antibody during hospitalization but showed a significant reduction during revisit. The remaining patients who had low titers during hospitalization, continued to have low titers during revisit. The authors used an exponential decay model to assess the decay rates of virus-specific antibodies and estimated that the predicted number of days before RBD-specific IgG titers decline to undetectable levels was 273 days. In contrast, three other studies reported persistent antibody titers for up to 3 months [55,69], or 4 months after diagnosis [70]. In the latter study by Gudbjartsson et al. in Iceland [70], they observed no decrease in the serum pan-Ig (IgG, IgM, IgA) against N protein and RBD up to 110 days post diagnosis, however when anti-S1 IgG was specifically measured, the OD values declined following a peak, after which, however, they remained at a stable level. In a study by Wajnberg et al. [55], antibody titers were measured in 121 donors at day 30 and day 82 post symptom onset, the average antibody titers in this group were shown to be stable over 3 months. A caveat of this study, however, is that they investigated the average of the titers of all patients, which as previously mentioned, is highly variable, and durable titers in some patients may have obscured declining titers in other patients. Another study by Isho et al. also reported a stable titer for 115 days [71], however, this study also used the average titers of a heterogenous group of patients, presenting the same caveat as the previous study. Lastly, a more recent study showed a slower decline in anti-RBD IgG titers over the course of 90 days, with relatively stable titers maintained [69]. Interestingly, this study also measured the durability of neutralizing antibodies, and observed a plateau of nAb titers up to 70 days post symptom onset, suggesting a relatively stable nAb titer. It is important to note that most of the patients in this study (93 %) required hospitalization, thus, in terms of disease severity, this group was skewed towards more severe disease.

**Table 1:** Studies on the durability of antibody responses to SARS-CoV-2.

Study	Disease severity of patients (number of patients)	Antibody response measured	Time points measured	Findings on durability of antibody responses
Ibarrondo et al., *NEJM* [67]	Mild (34)	Anti-RBD binding IgG	Titers measured once at day 37 and then at day 86 post symptom onset.	This study observed a decrease in titers between days 37 and 86 post onset of symptoms (a slope of -0.0083log_10_ per day). This corresponds to a half-life of antibodies to be 36 days, indicating a rapid decline.
Gudbjartsson et al., *NEJM* [70]	All disease groups (1237)	Anti-N and anti-S1-RBD pan-Ig, as well as anti-S1 IgG measured (binding titers)	Titers measured up to 110 days post diagnosis	This study observed no decrease in the pan-Ig anti-N and anti-S1 RBD serum titers. However, there was a decrease in the anti-S1 IgG OD values following the peak, after which the titers stabilized.
Long et al., *Nature Medicine* [66]	Asymptomatic (37); Symptomatic (37)	Binding and neutralization titers measured	Titers measured at 8 weeks post hospital discharge	This study observed a decrease in serum IgG titers (median decrease in titers was 71.1 % for the asymptomatic, and 76.2 % for the symptomatic patients). Neutralization titers decreased by 8.3 % in the asymptomatic group, and 11.7 % in the symptomatic group. Lastly, 40 % of asymptomatic became seronegative, while only 12.9 % of the symptomatic patients became seronegative.
Ma et al., *Sci. Chi. Life Sci*. [68]	Severe and moderate (27)	Binding IgG, IgM, IgA measured, cut-off-index reported	Titers measured at 28−99 days after discharge	This study observed a significant reduction in all binding antibody titers in all, but one, of the patients examined. They estimated the time at which antibodies will no longer be detectable to be 273 days.
Wajnberg et al., *Science* [55]	All disease groups (121)	Binding anti-spike protein IgG	Titers measured once at day 30 and then at day 82 post symptom onset.	This study found only a slight drop in average titers (from GMT 670 to 642) in the individuals screened, concluding that antibody titers are stable over time.
Isho et al., *Science Immunology* [71]	All disease groups (496 for serum, 90 for saliva)	Binding anti-spike and anti-RBD IgG, IgA and IgM titers in saliva and serum	Titers measured over the course of 115 days (binned into groups of 15 days)	This study observed a stable average level of anti-spike IgG in serum and saliva, with decreases in IgA and IgM. Antibody titers reported as relative ratios to a pool of positive controls.
Iyer et al., *Science Immunology* [69]	93 % of patients hospitalized (343 patients total)	Binding anti-RBD titers and neutralization titers	Titers measured up to 90 days post symptom onset	This study found a relatively stable serum IgG title in most of the patients, while IgA and IgM titers declined. nAb titers also plateaued up to 70 days post symptom onset.

**Fig. 5:**
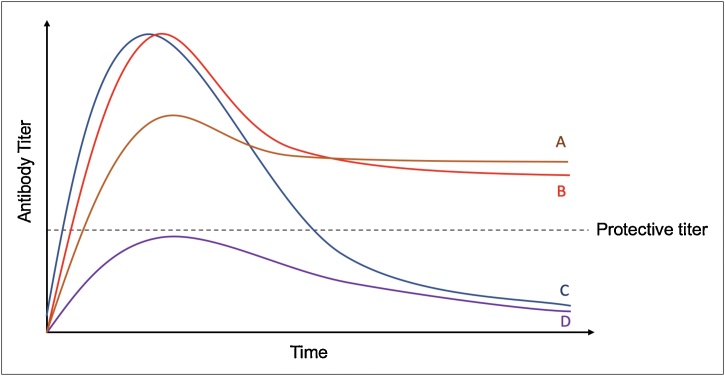
The durability of antibody responses over time in 4 different infection/vaccination scenarios.

In considering durability of antibody responses, as shown in Fig. 5, antibody titers following infection or vaccination can follow four different scenarios. In scenario A, antibody titers peak after disease onset, and gradually decline to a stable plateau above the protective threshold. In scenario B, although high peak titers in acute infection might not form, these titers decline only slightly, and remain above the protective threshold long term. In scenarios C and D, regardless of the peak antibody titers in the acute phase, long-term antibody responses are not generated. In addressing the longevity of antibodies to SARS-CoV-2, a practical definition of durability is the antibody titer after a given timepoint post resolution of infection, and the response can be considered durable if at the timepoint of interest, there are antibodies present above the protective titer. However, given the relatively short time that has elapsed since the emergence of SARS-CoV-2 infections, it is difficult to get a clear sense of what the durability of the response is. It is noteworthy that several studies report a decline in antibody titers, however as Fig. 5 curve A suggests, a steep slope of decline does not always imply poor durability, and a durable protective antibody titer can be achieved regardless of how steep the decrease from the peak titer was (when comparing curves A and B). In fact, in the Ma et al. study [68], the patients with higher initial titers had a much steeper decrease compared to patients with low acute phase antibody titers, which essentially remained stable. Thus, how durable antibodies are is more complex than the slope of decline following the initial infection and further longitudinal studies will need to be performed, in order to address the durability question precisely. Importantly, however, the initial evidence of the waning antibody titers indicates that SARS-CoV-2-induced humoral immunity will not be as durable as that of measles, mumps, chickenpox and other viruses, but is more likely to follow a similar pattern to SARS-CoV-1, where antibodies against the virus eventually wane after 2 years [56].

In summary, although there is emerging evidence on the limited durability of antibody responses to SARS-CoV-2, the extent to which neutralizing antibody titers decline remains less known. Thus, it is not clear how durable the functional antibody response to SARS-CoV-2 infection is. To date, only two of the studies has assessed the durability of neutralizing antibody titers, with one study observing a decrease in neutralizing activity of about 8.3 % in the asymptomatic group, and 11.7 % in the symptomatic group [66], and another showing a relatively stable nAb titer [69]. Compared to the decrease observed in the binding titers, the nAb titer decline is less sharp, indicating that low binding titers at late time points following recovery may not imply low neutralization activity. Furthermore, although the waning antibody titers observed in some of the studies could be concerning, it is important to note that a decline in serum antibody levels after the peak of infection is it to be expected, and what is more important is whether a persistent protective titer of antibodies can remain in the serum of recovered patients.

To sum up, there is significant evidence for seroconversion of COVID-19 patients, however, the binding antibody titers, and more importantly, the neutralizing antibody titers are highly variable between individuals. Moreover, the magnitude of neutralizing antibody response required for protection is currently not known. Lastly, there is emerging evidence for the limited durability of binding antibody titers, although the extent to which neutralizing antibody titers decline remains unknown. Consistent with the notion of short lived antibody responses, is recent work by Kaneko et al., showing loss of Bcl-6+ T-follicular helper cells and germinal centers in post-mortem analysis of the lymph nodes and spleens of acute SARS-CoV-2 infection, thus providing a mechanistic explanation for the limited durability of antibody responses in coronavirus infections [72].

### 3.4 T cell responses in SARS-CoV-2 infection

In addition to the antibody responses observed in convalescent COVID-19 patients, albeit at varying titers, recent studies have examined the antigen-specific T cell responses in infected patients. T cell activation has been reported in multiple studies in almost all infected individuals at both the acute and memory phases of infection [34,42,[73], [74], [75], [76], [77], [78], [79]]. However, the exact role of both CD4 and CD8 T cells in disease progression or protection is yet to be understood. In the acute phase of infection, robust CD8 T cell activation and proliferation has been observed, with increased proportions of terminally differentiated effector T cells in severe patients [42,75]. In addition, some studies report expression of exhaustion markers on CD8 T cells in patients with severe disease [75], with one study reporting functional exhaustion of T cells isolated from these patients [80]. Interestingly, CD8 T cells are not present in all infected patients and ∼20 % of patients did not have substantial CD8 T cell activation [42]. CD4 T cell activation has also been observed at the acute phase of infection, with some studies reporting the expression of exhaustion markers as well [42]. Furthermore, several studies observed a T_H_1 signature of CD4 T cells in COVID-19 patients [33,81], although in one study T_H_17 signatures were reported [82]. In a recent study of antigen-specific CD4 and CD8 T cell responses in acute and convalescent COVID-19 patients, a substantial Th1 and T_FH_ fraction was observed, with little Th2 or Th17 responses [83]. The individual contributions of CD4 and CD8 T cell responses in COVID-19 disease progression is not known. Although, there is some evidence for aberrant activation of T cells in severe disease [80,84], as stated above, antigen specific Th1 and T_FH_ responses could be detected in COVID-19 patients, and both arms of cellular immunity can contribute to an antiviral response [83]. Interestingly, this study observed that while nAb titers were not associated with decreased disease severity, antigen-specific CD4 and CD8 T cells responses were. This is consistent with the notion that T cell responses in infected patients can have a protective function. Antigen-specific CD8 T cells directly target virus-infected cells, while Th1 polarized CD4 T cells have the potential to activate CD8 T cells and monocytes to combat virus-infected cells in tissues. In addition, T_FH_ cells are necessary for germinal center responses and the formation of high quality humoral immune responses. Consistent with this, multiple studies have observed a correlation between CD4 T cell responses and binding antibody titers [76,78,79,85]. Thus, T cells can have a protective function both via direct elimination of infected cells, and via activation of other leukocytes and enhancement of humoral immune responses.

During the memory phase, it was shown that all convalescent COVID-19 patients have antigen-specific CD4 T cells, and about 70 % of the patients have antigen-specific CD8 T cells [76,77,79]. The magnitude of CD4 T cell responses in one study, as measured by expression of OX40 and CD137 upon stimulation with overlapping peptides, amounted to ∼0.5−1.0%. For CD8 T cells, the percentage of IFN-γ^+^ cells post-stimulation was at an average of ∼0.1 % with quite a lot of variability amongst patients [76]. In addition, seronegative patients, who were family members of COVID-19 infected patients or were themselves mildly or asymptomatically infected with COVID-19, were also reported to have T cell responses against SARS-CoV-2 antigens [79]. Importantly, in seropositive individuals, CD4 T cell responses were positively correlated with anti-spike and anti-RBD IgG and IgA [76,78,79,85]. The targets of the T cell responses in COVID-19 patients is also interesting; unlike other coronaviruses, where the spike protein is thought to be the major dominant T cell epitope [86], in the case of SARS-CoV-2, the spike, nucleocapsid as well as matrix proteins are co-dominant CD4 T cell epitopes, and CD8 T cell targets include both the spike and M protein [76]. Considering that most of the current vaccine candidates include spike protein as the target antigen, the specificity of the vaccine-induced T cell immunity will be different from that induced by natural infection, and the impact of this on protection remains unclear. Thus, future vaccine efforts should be aimed at assessing the dominance hierarchy of the different T cell epitopes in the spike versus N versus MP proteins in terms of their relative contributions to stimulating protective T cell immunity.

Interestingly, T cell responses were detected in healthy subjects with no history of COVID-19 [74,76,77]. CD4 T cell responses were observed in ∼50 % of healthy unexposed donors, whereas CD8 T cell responses were seen in ∼20 % of the patients [76]. The magnitude of T cell responses in some of the unexposed patients seemed to reach that of COVID-19 patients, however, on average the percentage of responsive T cells was lower in these healthy controls. Based on the epitopes to which these T cells are responding to, it has been proposed these are cross-reactive T cells that were generated with a prior infection with the common cold coronaviruses. However, this remains to be proven and the role of these T cells in COVID-19 immunity are yet to be clarified. Indeed, although memory T cells are apparent in recovered patients, and even some unexposed healthy individuals, their potential role in protection against reinfection is yet to be understood, and whether the observed T cell immunity is protective, pathological or inconsequential for protection, remains to be clarified. If protective, T cells can not only directly induce killing of virus-infected cells, but also affect the overall immune response in the tissue, orchestrating a multidimensional anti-viral response, facilitating the activation of innate cells, induction of potent neutralizing antibodies and recruitment of other leukocytes to the site of inflammation [87]. These could be the T cell functions to be harnessed in vaccine design to elicit protection against reinfection. However, the durability of T cell memory is also not understood due to the recency of infections, although, experience from a 6-year follow-up study of SARS-recovered individuals has shown long-lasting T cell responses even when antibody responses are no longer detectable [88]. If this holds true for SARS-CoV-2, then it is highly likely that durable T cell responses will be present.

## 4 Vaccine-induced immunity against SARS-CoV-2

As mentioned above, a plethora of vaccine platforms is currently being used in developing COVID-19 vaccines (Fig. 2). The more established platforms, such as inactivated viruses and recombinant protein-based vaccines, have been used for vaccines against various infectious diseases, such as influenza, and are currently being tested for SARS-CoV-2 as well (for example, vaccines from Novavax, Sinovac, Sanofi and GSK). The newer mRNA vaccine platforms have not been licensed for use in humans yet, however, they can be made very rapidly. Indeed, soon after SARS-CoV-2 was isolated and sequenced in January 2020, the entire process starting from antigen design to IND review and clinical trial took an astounding 63 days [89], which is unprecedented in the entire history of vaccinology. Lastly, adenovirus-based vaccine candidates (such as the ones from Oxford University and CanSino) were already being tested as candidates for MERS vaccine, and adapting them to SARS-CoV-2 proved to be quick and efficient. However, this method has a disadvantage due to the presence of anti-vector immunity against Ad5 in the human population, which can potentially affect the vaccine immunogenicity and lead to disease enhancement [90]. Below, we discuss the current data available for the vaccine candidates in human trials with a mention of some non-human primate studies of other candidates.

### 4.1 Antibody responses induced by vaccination

#### 4.1.1 Clinical trial results

As mentioned above, neutralizing antibodies are induced in the majority of COVID-19 patients. In addition, as mentioned above, preliminary evidence suggests a key role for neutralizing antibodies in protecting against infection in rodents. In line with this, the majority of the current vaccine candidates are being evaluated for their capacity to induce humoral immunity (measured by the magnitude of binding antibodies to the coronavirus spike protein), as well as the quality of the antibody responses (focused on the neutralization capacity of these antibodies). A major target is the coronavirus spike (S) protein, particularly its receptor-binding domain (RBD), since binding to this domain prevents the conformational change required by the virus to efficiently bind ACE2 and enter human cells. Thus, most studies measure anti-RBD and anti-S titers in the sera of immunized patients or animals. However, the ELISA titers reported differ from study to study, where some studies report endpoint titers, whereas others report midpoint titers, making comparisons between vaccine candidates challenging. In addition, in order to evaluate the induction of nAbs by different vaccine candidates, in some studies, *in vitro* virus neutralization assays are performed using pseudoviruses, while in others - live SARS-CoV-2 isolates. Thus, in drawing comparisons between vaccine candidates these factors need to be taken into consideration. Many of the current reports include serum samples from convalescent patients, as a reference point for comparing vaccine-induced antibody titers. However, as previously mentioned, patients vary greatly when it comes to their antibody titers, thus, a mere comparison to the mean convalescent titers is not helpful. Comparing the vaccine-induced titers with serum titers from hospitalized patients (severe COVID-19 cases), however, can be informative, considering these are the patients with the highest antibody titers observed from natural infection. Below, we review the human trial data from the vaccine candidates currently in clinic, and for candidates for which human clinical data has not be reported, we review the non-human primate data. Table 2 summarizes the outcomes of Phase I/II clinical trials of 9 vaccine candidates that have published their results thus far. Of the RNA vaccines being currently tested in human trials, Moderna’s mRNA-1273 construct, which encodes the spike protein S-2 P antigen, showed promising results from a preliminary report of a phase 1 clinical trial in humans comparing three doses (25, 100 and 250 micrograms) of mRNA-1273. The vaccine was given as a two-dose vaccine with immunizations 28 days apart, and after the second vaccination, high binding antibody titers for spike protein were reported, which increased with the dose (Table 2) [2]. The serum-neutralizing activity was detected in all participants evaluated, with values generally similar to those in the upper half of the distribution of a panel of control convalescent serum specimens [2]. Interestingly, systemic adverse events were more common after the second immunization, particularly with the highest 250 μg dose group, where three participants (21 %) reported one or more severe adverse events.

**Table 2:** Summary of clinical trial results of COVID-19 vaccine candidates.

Another RNA vaccine candidate– BNT162b1, a lipid nanoparticle formulated mRNA vaccine encoding the trimeric SARS-CoV-2 spike protein receptor binding domain (RBD), from BioNTech/Pfizer – was tested in two dose-escalation phase 1 studies of 18–55 year-old healthy adults, randomized to receive 2 doses separated by 21 days [3,91]. In both clinical trials, local reactions and systemic events were generally mild, but in the first trial, the second 100 ug dose (highest tested) was not administered because of reactogenicity and a commensurate enhancement of immunogenicity relative to the lower (30 ug) dose. Similar to the Moderna study, there were enhanced RBD binding and neutralizing titers in sera after the second immunization. Geometric mean neutralizing titers reached 1.9–4.6-fold that of a panel of COVID-19 convalescent human sera [3]. Similarly, in the second phase 1/2 trial with the same vaccine [91], immunization induced a strong, dose-dependent antibody response 21 days after primary immunization, which increased over 10-fold following the second immunization. Notably, the titers detected were at least 5-fold higher than the average titers measured in convalescent sera from COVID-19 patients in this study. Neutralizing antibodies could be detected, albeit at low titers, at 21 days after priming, and following the boost, there was a substantial (∼5-fold) increase in the neutralization antibody titers, similar to that observed in the U.S study. In addition to these effects on the antibody response, impressive T cell responses were induced in the majority of vaccinees, with remarkably high RBD-specific CD4 and CD8 T cell responses, comparable in magnitude with memory responses observed against CMV, influenza and tetanus toxoid. The frequency of RBD-specific CD8 T cells was remarkably high, with several vaccinees having frequencies as high as 5% RBD-specific CD8 or CD4 T cells.

Four viral vector vaccines have also reported the results of their clinical trials (Table 2). The study of CanSino Ad5 construct increased mean titers of anti-RBD, as well as neutralizing antibodies upon immunization, however, the overall magnitude of the response was not as potent compared with some other vaccine candidates [4] (Table 2). This study did not contain a group of convalescent sera for comparison to the neutralization titers observed with the vaccination, thus, making it difficult to put it into context of other candidates with regards to its potency. Interestingly, this paper also observed a positive correlation with anti-RBD antibodies and neutralization titers. The second viral vector vaccine candidate is ChAdOx1 nCoV19 from Oxford University, a chimpanzee adenoviral vector encoding the sequence of the spike protein [5]. This vaccine, also called AZD-1222, is being developed jointly by Oxford University and AstraZeneca. Unlike the case with Ad5, humans have a low seroprevalence of ChAd, therefore this candidate does not have the potential complications of pre-existing immunity associated with Ad5 vectors. This study contained two groups of patients – a larger group that received a single shot of the vaccine, and a group of 10 participants that received a boost at day 28. The average neutralization titers were higher for the prime-boost group, which is in line with the previous reports in NHPs that boosting enhances the immunogenicity of the vaccine [92]. The antibody titers induced looked close to (if not slightly less than) the average convalescent samples, however, the exact titers for convalescent patients were not provided, making it difficult to compare. Interestingly, this vaccine also induced significant T cell responses, measured by IFN-γ ELISPOT assays, still detectable at late time points post vaccination (Table 2). The third viral-vector vaccine candidate from Russia, based on a prime boost vaccination of recombinant Ad26 at day 0, and rAd5 at day 21 (both expressing the S protein), was tested in a phase 1/2 trial [6]. This vaccine was similar in immunogenicity, and maybe even more potent, when compared to the other viral vector vaccine candidates, with regards to the average binding and neutralizing antibody titers measured. It is important to note that the binding antibody titers were about tenfold higher in vaccinated patients compared to convalescent patients (only moderate and severe patients included in convalescent group), however the neutralizing antibody titers were not significantly different from convalescent patients. In addition, both CD4 and CD8 T cell proliferation, as well as IFNγ production, were observed in PBMCs post stimulation with antigen. Only mild systemic and local adverse events were observed in participants following vaccination. Lastly, the Ad26 viral vector vaccine developed by Janssen/Beth Israel also showed immunogenicity in terms of inducing anti-spike protein binding antibodies and neutralizing antibodies, although the titers for both of these determinants were lower than the average titers observed from human convalescent sera [7]. CD4 and CD8 T cell responses were also observed with a single dose of this vaccine. Importantly, this trial has yet to release data from the prime-boost immunization groups, thus, more information is needed to fully assess the immunogenicity of this candidate, since the second immunization might significantly boost the nAb titers, as seen with other vaccine candidates. Thus, all of the viral vector vaccine candidates seem to be similar in terms of both safety and immunogenicity, however, these seem to be slightly inferior to the RNA-based vaccine candidates and the adjuvanted protein-based vaccines (discussed below).

In addition, two inactivated virus vaccine candidates from China have released Phase I clinical data (Table 2) – the inactivated vaccine candidate from Wuhan Insititute of Biological Products/Sinopharm [10], and CoronaVac from Sinovac [9]. Both of these vaccine candidates showed immunogenicity, with the induction of binding and neutralizing antibodies in the majority of volunteers. However, convalescent sera were not analyzed in these studies, thus, a comparison cannot be made between these candidates and others, although in the study of CoronaVac, the authors referred to an earlier assessment of convalescent nAb titers by the same assay, which was higher than the titers observed in inoculated individuals.

Lastly, the first recombinant protein vaccine candidate (NVX-CoV2373, Novavax) was tested in Phase I clinical trial [8] (Table 2), and a key highlight of this study is the strong effect of Matrix M, a saponin based adjuvant, used along with the different doses of antigen. Without the use of adjuvant, the 25 ug protein antigen group yielded anti-spike antibodies of titers much lower than that observed in convalescent patients. However, the addition of Matrix M significantly increased both anti-spike IgG, (reaching a titer of 60,000), as well as neutralizing antibody titers (a titer of >3300 as measured by a liver virus microneutralization assay). These titers are much higher than the titers seen in asymptomatic or outpatient-treated COVID patients but are similar in magnitude to hospitalized COVID-19 patient serum titers. In addition, this study also reported that total anti-spike IgG correlated with live-virus neutralization titers – a trend seen in multiple vaccine studies thus far.

#### 4.1.2 Animal model results

The key question with all the COVID-19 vaccine candidates is how effective they are at preventing or controlling infection. Phase 3 efficacy trials are currently underway with several vaccines including the Moderna, BioNTech/Pfizer, Oxford/AstraZeneca vaccines, and are expected to yield answers to this question. In the interim however, the results of studies in nonhuman primates offer some clues. In addition to the promising human clinical trial results, many other vaccine candidates have reported induction of SARS-CoV-2 spike-/RBD-binding, as well as neutralizing antibodies, in their preclinical studies. The preclinical results from vaccine candidates that have reported human trial results are summarized in Table 3, while other candidates are reviewed below. The inactivated SARS-CoV-2 vaccine developed by SinoVac [93] in mice has shown high binding and neutralization titers. Similarly, immunization of rhesus macaques with this inactivated vaccine also resulted in the induction of anti-RBD binding as well as neutralizing Abs. Importantly, this study showed protection from infection with SARS-CoV-2 in NHPs, providing evidence for the potential protective capacity of this vaccine.

**Table 3:** Summary of preclinical trial results of COVID-19 vaccine candidates in clinical trials.

Several nucleic acid vaccine platforms, that were not mentioned previously, have also shown induction of humoral immunity in pre-clinical studies. Immunization of NHPs with a DNA construct expressing versions of the spike protein (including full-length S, S protein with certain domains deleted, as well as trimerized RBD) induced nAb titers comparable to convalescent humans, and provided with protection against reinfection, as measured by lower viral RNA observed in the BAL and nasal swabs of animals challenged with the virus) [64]. In addition, the INO-4800 construct from Inovio Pharmaceuticals, when tested in mice and guinea pigs, showed an increase in the titers of anti-S and anti-RBD antibodies, but the overall endpoint titers seem to be slightly lower than the titers observed in the previously-mentioned studies of other vaccine candidates [94]. Other candidates, such as a replicon RNA vaccine [95], also showed induction of antibodies when immunized in NHPs, with nAb titers slightly lower than that of convalescent patients measured in this study. Lastly, in addition to the viral vector vaccine candidates mentioned above, the Modified Vaccinia Ankara vector encoding either the full spike protein or the S1 subunit of spike protein [96] showed induction of Ab responses, with the MVA-S construct inducing higher binding and neutralization titers of anti-RBD antibodies compared to MVA-S1. Importantly, this study showed a strong positive correlation between the anti-RBD binding titers and virus-neutralization.

Taken together, there is convincing evidence that many of the current vaccine candidates induce moderate to strong antibody responses. Furthermore, the induction of antibodies seems to correlate with virus neutralization and protection against challenge (discussed below) in the studies that used challenge models. Undoubtedly, the humoral immune arm is crucial for mediating anti-viral protection and in the pursuit of an efficacious COVID-19 vaccine we must understand what a successful vaccine candidate should entail, particularly with regards to the humoral immune response: the choice of antigen to which antibodies are to be induced against, the necessary magnitude of these antibodies, and their neutralization capacity are all important factors. However, in pursuing the COVID-19 vaccine, we must look beyond neutralizing antibodies as our only correlate of protection, since, neutralizing antibodies alone may not be sufficient and durable, and low titers of neutralizing antibodies coupled with cellular immunity may induce more durable protection against reinfection.

### 4.2 T cell responses induced by vaccination

As mentioned above, antibodies have long been considered the classical correlate of protection, thus, many of the vaccine studies do not focus on the T cell immunity induced following vaccination. T cell responses are usually measured by a cytokine ICS assay or ELISPOT following a stimulation with a SARS-CoV-2 spike protein peptide pool. As summarized in Table 2, Table 3, several vaccine candidates were tested with regards to their capacity to induce T cell responses. The mRNA vaccine candidate mRNA-1273 from Moderna showed induction of Th1 and Tfh responses in NHPs, but did not find any evidence of Th2 or CD8 T cell responses [65]. Similarly, Th1 CD4 T cell responses were observed in human participants immunized with the 100 ug dose [2]. Interestingly, the mRNA vaccine candidate from Pfizer induced significant CD4 and CD8 T cell responses in humans [91]. In addition, the viral vector ChAdOx1 vaccine from Oxford University reported evidence of both Th1 and CD8 T cell responses in NHPs, and IFNy-producing T cells in their human trial [5]. Thus, the mRNA and viral-vector-based platforms may be beneficial for the induction of T cell responses. In contrast, no T cell responses were observed for the inactivated virus candidate from Sinovac in their preclinical studies with mice and NHPs [93], and T cell responses were not assessed in the human Phase I clinical trials of both inactivated vaccine candidates [9,10]. Interestingly, the recombinant protein vaccine from Novavax combined with Matrix M adjuvant showed multifunctional CD4 T cell responses, although CD8 T cell responses were not measured [97], and this effect was not observed without the addition of the adjuvant. In the absence of T cell tetramers against SARS-CoV-2, it is difficult to further study antigen-specific T cell function beyond *in vitro* cytokine production assays. Of particular interest would be understanding the memory subsets induced following vaccination (circulating versus tissue-resident) and the helper T cell polarization states (Th1, Th2 vs Th17). Nevertheless, the results from some of the current studies show promising CD4 Th1 responses, and in some cases CD8 T cell responses, but the extent to which this aspect of vaccine-induced immunity correlates with protection is not clear and remains to be further investigated.

### 4.3 Vaccine-induced protection

The efficacy of vaccines is assessed in phase 3 clinical trials, and the results of these trials for some of the COVID-19 vaccine candidates are expected in the coming weeks or months. In addition, vaccine-induced protection in nonhuman primate challenge studies has been assessed by measuring of viral load via PCR in nose swabs and broncho-alveolar lavage fluid (Table 3). For example, the study of the mRNA vaccine candidate from Moderna measured viral loads in BAL and nose swabs post challenge of NHPs. In this study, they found that while LRT protection can be achieved with all of the doses, only partial URT protection can be seen, and only in the higher dose groups (100ug) [65]. The study of ChAdOx1-nCov-19 vaccine from Oxford also observed similar results, with protection seen in the LRT, but viral RNA present in nose swabs [92]. Although LRT protection is important, and can imply reduced disease severity in vaccinated individuals, the results from these studies suggest that upon infection, vaccinated individuals can still remain infectious to others. In contrast, the inactivated virus vaccine from Sinovac, as well as the protein vaccine from Novavax, showed significantly reduced viral loads in both URT and LRT [93,98] (Table 3), indicating that both reduced disease severity and virus transmission can be achieved by these vaccines.

Thus, there is recent evidence from NHP studies that some of the current COVID-19 vaccine candidates can prevent LRT, and in some cases, even URT infection, which is promising. However, it remains to be yet identified what the correlates of protection are for both URT and LRT protection, although neutralizing antibodies did correlate with reduced viral load in the BAL in one study [64]. It is interesting, however, that some vaccines induce protection in LRT, but not URT, suggesting that the correlates of protection for each of these sites is different. In fact, for influenza infections, it has been shown that while IgA seems to be the major protective pathway for the URT, IgG is more important for LRT protection [99].

Although many of the current vaccine candidate studies report anti-spike/RBD binding and neutralizing IgG responses, with some studies also presenting data on antigen-specific T cell responses, they fail to include many other aspects of immunity to vaccination, such as mucosal antibody responses, T cell memory subset differentiation in response to vaccination and the potential for induction of trained immunity of the innate compartment. Below, we suggest some future considerations for vaccine development that could potentially result in vaccines that target a more synergistic and multi-faceted immune response.

## 5 The many immunological roads to protection against SARS-CoV-2

### 5.1 Tissue resident memory T cells

Tissue-resident memory T cells (T_RM_) represent a recently discovered subset of T lymphocytes that are widely distributed in tissues and emerging as important mediators of immunity to many pathogens. Localized in mucosal and non-mucosal tissues, T_RM_s are poised for rapid deployment of their effector function upon secondary exposure to pathogens [100]. In addition, T_RM_ recruit other immune cells to the site of infection, thus mobilizing multiple host defense mechanisms in the tissues [100,101]. These immunological features make T_RM_s an attractive target for vaccinologists and learning how to induce T_RM_ and maintain them in tissues represents an important goal in vaccinology.

As already mentioned, the major focus in vaccine design has always been on inducing humoral immunity, particularly neutralizing antibodies. However, recent studies have begun to highlight the importance of the potential interactions between multiple arms of the immune system in vaccine induced protection [87,102]. In our recent study with HIV vaccination, we observed that the presence of tissue resident memory CD8 T cells lowered the threshold of neutralizing antibody required for protection against the re-challenge, indicating that there is potential synergy between the cellular and humoral components of protection [87]. In addition, mucosal resident CD4 T cells in the female reproductive tract, have been shown to recruit memory B cells upon rechallenge, mediating enhanced protection [102].

Thus, besides direct antiviral functions, resident memory T cells can influence the humoral immune response, mediating protection against reinfection both directly and indirectly. Understanding the role of T_RM_-mediated immunity for protection against reinfection, and harnessing this arm of the immune response, should be a major goal of COVID-19 vaccine research and development for many reasons. First, T cell immunity can target the more conserved regions of the virus which are often not accessible to antibodies, resulting in a potential cross-protection against multiple coronaviruses. As it was observed in one study, an intranasal vaccination of mice with Venezuelan equine encephalitis replicons (VRP) encoding the SARS-CoV-1 nucleocapsid protein CD4 T cell epitope, resulted in the induction of airway memory CD4 T cells that not only protect against reinfection, but also yield cross-protection against MERS [103]. Lastly, reactivation of T cells in the tissue upon reinfection can induce a robust activation and recruitment of other leukocytes, orchestrating a more robust and synergistic protection.

### 5.2 Use of adjuvants to enhance vaccine-induced immunity

Adjuvants are substances that are added to vaccines to enhance the magnitude and durability of antigen-specific antibody and T cell responses. Adjuvants trigger various pathways of innate immune activation, such as pattern recognition receptor signaling and cell death, which in turn results in activation of innate and adaptive immunity [104]. Currently, adjuvants that have been used in the clinic include insoluble aluminum salts such as Alum, as well as oil-in-water emulsion adjuvants such as MF59 and AS03 (used for the pandemic influenza vaccines), and TLR agonists such as CpG1018 (used in the Hepatitis B vaccine) [105]. Currently, over 10 developers are planning to develop adjuvanted vaccines [106]. As mentioned previously, the Novavax recombinant protein vaccine combined with Matrix M adjuvant induced robust humoral responses, as well as some Th1 CD4 T cells in immunized patients [97]. The strong effect of the adjuvant in this study highlights the potential of adjuvants in inducing robust immune responses. Moreover, adjuvants have been shown to increase the durability of humoral responses [104]. A striking example of this concerns our recent study of HIV antigens adjuvanted with a TLR7/8 agonist adjuvant called 3M-052 showed the induction of HIV envelope-specific long-lived plasma cells and durable antibody responses in non-human primates [107]. Thus, adjuvants present a great potential for inducing a higher magnitude and durability of humoral responses, well as dose-scaling which could accelerate vaccine production significantly. Therefore, studies comparing the efficacy of various adjuvants are warranted, and the capacity of each of these adjuvants to induce robust humoral and cellular immunity and protection, when combined with SARS-CoV-2 antigens, are currently being tested in nonhuman primates.

### 5.3 Harnessing trained immunity for broader antiviral protection

The phenomenon of immunological memory has historically been attributed to B and T lymphocytes. However, recent work has highlighted the concept of trained immunity – the epigenetic reprogramming of innate cells in response to particular stimuli [108]. This epigenetic reprogramming has been observed post vaccination with Bacillus Calmette-Guérin (BCG), but whether or not such boosted innate immune profile would be beneficial or harmful for SARS-CoV-2 should be carefully investigated [109]. In one study, there did not seem to be a statistically significant difference between the SARS-CoV-2 infection rates of people that received the BCG vaccine versus those who did not [110], however, it is possible that the effect of BCG vaccination in inducing elevated innate responses is dependent on the recency of the vaccine administered, thus, a more careful examination of the effect of BCG vaccine is warranted. In this context, recent work by Mihai Netea and colleagues on a phase 3 ACTIVATE clinical trial assessed 198 elderly patients, who received BCG or placebo vaccine at hospital discharge, and were followed for 12 months for new infections of any kind [111]. At interim analysis, BCG vaccination significantly increased the time to first infection (median 16 weeks versus 11 weeks for placebo), as well as the incidence of new infections (42.3 % for placebo versus 25 % for BCG).

## 6 Systems vaccinology: accelerating the vaccine testing pipeline

The traditional vaccine developmental paradigm that involves preclinical testing in animal models, followed by clinical testing in phase 1, 2 and 3 clinical trials, has been very successful in enabling the development of all the licensed vaccines. However, this developmental process can normally take several years and can cost up to $500 million [11,12]. The United States government launched a Manhattan Project Style project termed Operation Warp Speed, aimed at delivering 300 million doses of a safe and effective COVID-19 vaccine by January 2021, as part of a broader strategy to accelerate the development, manufacturing and distribution of COVID-19 vaccines and other countermeasures such as therapeutics and diagnostics [112]. Led by the U.S. Department of Health and Human Services and the Department of Defense in partnership with the private sector, Operation Warp Speed has galvanized the development of COVID-19 vaccines and progressed at a pace that is unprecedented in the history of vaccines. For example, on January 13th, two days after the Chinese authorities shared the genomic sequence of the virus, scientists at Moderna and the NIH finalized the sequence for the mRNA1273 vaccine, and on March 16th the NIH announced that the first subject in the phase 1 clinical trial had received the vaccine. Thus, it took a mere 63 days from sequence selection to phase 1 clinical trial [89]. As of September 20th, 2020, the phase 3 clinical trial of the mRNA1273 vaccine, as well as eight other candidate vaccines are ongoing. The rapid progress from concept to phase 3 trials, and hopeful licensure within a few months, is unprecedented in the history of vaccines.

What made the warp speed development of vaccines possible? One important factor was novel technologies. For example, the mRNA technology facilitated the rapid design and synthesis of the candidate vaccine, immediately upon knowledge of the genomic sequence of the virus. However, an additional important factor was that Operation Warp Speed, as well as other companies, were willing to assume the financial risk by starting the manufacturing of the candidate vaccines at industrial scale, even before the efficacy and safety results of the phase 3 clinical trials became known.

**Fig. 6:**
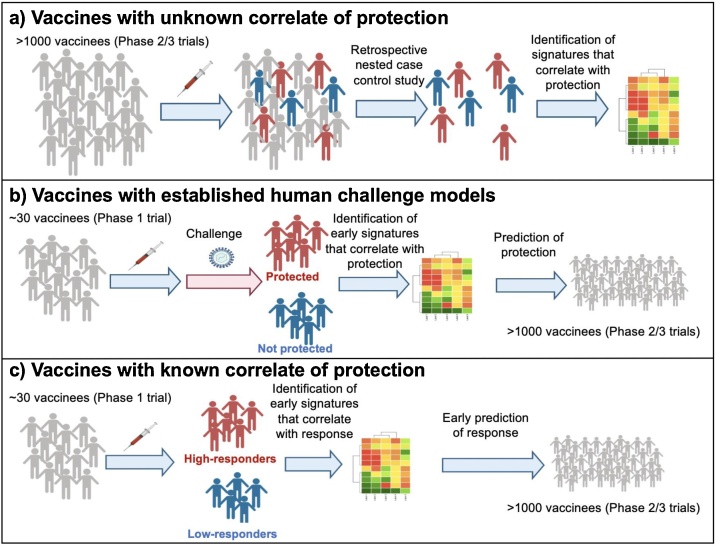
Systems vaccinology approaches for accelerating the vaccine-testing pipeline.

Clearly, the warp speed vaccines effort represents unprecedented and laudable progress in the development of vaccines against COVID-19 vaccines. Yet it also presents many challenges and opportunities [129]. One major challenge is that at present we do not have a correlate of protection, so the efficacy of all vaccine candidates will need to be assessed in large phase 3 trials, involving tens of thousands of people. Clearly the costs involved would be prohibitive. Defining a correlate of protection will permit the rapid assessment of multiple vaccine candidates in smaller, phase 1 or 2 trials. Most of the current vaccine candidates are being developed on the premise that the magnitude of neutralizing antibody response will be a determinant of protection. Therefore, defining an antibody correlate – a threshold of neutralizing antibody titer – that is necessary for protection is a first step. However, as discussed above, whilst neutralizing antibodies are the main mechanism by which vaccination prevents infection, the immune system has multiple mechanisms of defense against an infection, and T cells and the innate immune system can provide complimentary mechanisms. Indeed, different COVID-19 vaccines may trigger distinct mechanisms of immune protection. Therefore, performing a comprehensive analysis of the immune response to vaccination will help define correlates of protection induced by different vaccines. In this context systems vaccinology approaches, have identified molecular signatures induced within a few days (e.g. day 1, 3 or 7) of vaccination, or at baseline, that correlate with and predict the ensuing antibody and T cell responses, or protection against infection [113,114]. Systems vaccinology uses machine learning approaches to define early signatures that can be used to predict immune responses and protective immunity [113,114]. Such an approach has been used to define molecular correlates and predictive signatures of the immunogenicity of vaccines against yellow fever [115,116], influenza [[117], [118], [119], [120], [121]], malaria [122], Ebola [123] and other diseases. This approach has not only provided molecular signatures of vaccine immunity, but also yielded new mechanistic insights about the how vaccines induce immune responses [[124], [125], [126], [127], [128]]. Such a systems biological approach could conceivably be used in the COVID-19 vaccine effort to define early, or baseline, molecular correlates of the magnitude and durability of antibody responses or T cell responses, or protective immunity against SARS-CoV-2 infection. The latter can be ascertained in retrospective, nested, case-controlled studies, in which “banked” blood samples from a phase 3 trial can be analyzed to identify correlates of vaccine efficacy, as described previously [113] (Fig. 6a). In addition, systems vaccinology approaches can be used to define correlates of vaccine efficacy in CHIM of COVID-19 infection, similar to the studies undertaken with CHIM models of malaria infection [122] (Fig. 6b). Finally, knowledge of an immunological correlate of protection (e.g. threshold of neutralizing antibody, as could be determined from a phase 3 trial) will allow rapid screening of new vaccine candidates, in relatively small phase 1 trials (Fig. 6c). Here, systems based approaches offer a means to rapidly identify molecular signatures of immunogenicity, that exist at baseline, or that are induced only a few days after vaccination, thereby negating the need to wait several weeks before the onset of neutralizing antibody titers. Thus, a systems-level approach at investigating the immune response to coronavirus infection is warranted and will allow for simultaneous analysis of the multiple facets of the protective immune response. The identification of pathways that are correlated with protection can help inform us of the immunological mechanisms that are necessary for protection, and accelerate the vaccine testing pipeline, allowing for prediction of a vaccine’s future protective potential starting at phase 1 trials. Furthermore, identification of the necessary mechanisms would enable the design of new vaccine candidates that engage the desired pathways. Thus, systems vaccinology can help not only test current candidates but also guide the design of novel vaccine candidates and inform basic research regarding the immune response to SARS-CoV-2.

## 7 Concluding remarks

In the tragic eleven months since its emergence in December 2019, SARS-CoV-2 has galvanized immunology and vaccinology. The unprecedented speed with which COVID-19 vaccines are being developed and licensed (∼6 months, compared to the typical 10–15 years) [129], the rapid harnessing of innovative new technologies (e.g. mRNA vaccines and novel adjuvants), major public and private investments in COVID-19 immunology and vaccinology, and the world’s fascination with vaccines and immunology, has underscored the global imperative of vaccines. SARS-CoV-2 has forced us to reexamine the very fundamentals of the social, political, economic and scientific fabric with which our world has been built. It is up to us to learn from this tragedy such that, when we face our next microbial adversary, we will never again be where we are now. For us immunologists and vaccinologists, that means continuing the galvanic trajectory of discovery and translation, and redefining the way we conceive of, make, test, license and distribute vaccines.

## Disclosures

Bali Pulendran serves on the External Immunology Network of GSK, and on the Scientific Advisory Board of Medicago.
